# mRNA Levels of MAOA and 5-HT_**2***A*_ Receptor in Patients With Pathological Internet Use: Correlations With Comorbid Symptoms

**DOI:** 10.3389/fpsyt.2021.667699

**Published:** 2021-07-16

**Authors:** Mimi Qiu, Chenchen Zhang, Yu Dai, Lingrui Zhang, Yang Wang, Wei Peng, Yalin Chen, Chao Wen, Hui Li, Tianmin Zhu

**Affiliations:** ^1^College of Rehabilitation and Health Preservation, Chengdu University of Traditional Chinese Medicine, Chengdu, China; ^2^Department of Rehabilitation, Traditional Chinese Medicine Hospital of Longquanyi District, Chengdu, China; ^3^Department of Medicine, Leshan Vocational and Technical College, Leshan, China; ^4^College of Acupuncture and Tuina, Chengdu University of Traditional Chinese Medicine, Chengdu, China; ^5^Department of Rehabilitation, Zigong Fifth People's Hospital, Zigong, China; ^6^College of Medicine, Chengdu University, Chengdu, China

**Keywords:** pathological internet use, monoamine oxidase type A, 5-HT2A receptor, mRNA level, comorbid symptoms

## Abstract

**Objective:** Uncontrolled internet use may lead to the emergence of pathological internet use (PIU). PIU has become a global public health concern that can cause a range of psychotic symptoms, including anxiety, depression, and impulse control disorder. To date, we know very little about the principal biological factors related to PIU. Monoamine oxidase type A (MAOA) and serotonin (5-HT) 5-HT_2A_ receptor (5-HT_2A_R) play critical roles in the development of behavioural and drug addictions. Thus, the aim of this study was to measure the relative expression of mRNA of MAOA and 5-HT_2A_R in peripheral blood mononuclear cells (PBMCs) of patients with PIU and to determine the correlations between these biological indicators and the comorbid symptoms of patients with PIU.

**Methods:** In this study, the mRNA of MAOA and 5-HT_2A_R was detected using real-time PCR in PBMCs of the patients with PIU (*n* = 24) and healthy controls (HCs, *n* = 25). The relationship between the mRNA levels of MAOA and 5-HT_2A_R and clinical symptoms in patients with PIU was further investigated.

**Results:** MAOA mRNA in PBMCs was significantly upregulated in patients with PIU compared with that in HCs. mRNA levels of 5-HT_2A_R were not found to differ significantly between HCs and patients with PIU. Correlation analyses further revealed a significant positive correlation between the relative expression of MAOA mRNA in PBMCs of patients with PIU and the Young's Internet Addiction Test and Self-Rating Depression Scale scores.

**Conclusion:** The present study revealed upregulated expression of MAOA mRNA in patients with PIU and an association between the expression of MAOA mRNA and clinical symptoms of PIU, suggesting that the neurobiological changes may be similar between PIU and substance addiction. Additionally, this study demonstrated a potential association between comorbid symptoms and mRNA levels of MAOA.

## Introduction

Pathological internet use (PIU) is defined as a behavioural addiction, which involves increasing use of the internet for a long time to obtain satisfaction, surfing the internet longer than intended, irritability during deprivation from the internet, and excessive internet use leading to functional deficits (1). Because of high rates of prevalence and psychiatric comorbidity, PIU has become a serious public health concern. The features of PIU include loss of interest or enjoyment in otherwise pleasurable activities, accompanied by other comorbid psychiatric symptoms such as impulse control disorder, anxiety, depression, insomnia, and attention-deficit/hyperactivity disorder (ADHD) (2–5). Studies have shown that 23.3, 26.3, and 21.7% of patients with PIU suffer from anxiety disorder, depression, and ADHD, respectively (6). Many researchers have confirmed the aetiological mechanism of PIU, and considerable evidence is available regarding changes in the central and peripheral nervous systems in patients with PIU (7, 8). However, the exact physio-pathological mechanism of PIU remains unclear.

Diminished activity of the serotonin (5-hydroxytryptamine, 5-HT) system has been implicated in the emergence of addictive diseases (9, 10). One of the main receptors for serotonin is the 5-HT_2A_ receptor (5-HT_2A_R), which regulates different pathophysiological aspects, including mood, sleep, learning, and memory, of diseases of the nervous system (11–13). Intriguingly, considerable evidence has shed light on the importance of 5-HT_2A_R in the vulnerability and establishment of drug-related behaviours, suggesting a role of 5-HT_2A_R in transition to and maintenance of addiction (14, 15). In a previous positron emission tomography (PET) study, patients with PIU were found to have reduced serotonin 5-HT_2A_R in the left and right temporal cortex that was related to the low level of dopamine D_2_ receptors in the striatum (16). Moreover, studies have identified that abnormal levels of neurotransmitters such as serotonin (5-HT), norepinephrine (NE), and dopamine (DA) in individuals are closely associated with comorbid psychiatric symptoms of PIU (17–19).

Monoamine oxidase A (MAOA) is a mitochondrial enzyme that catalyses the degradation process of various amine neurotransmitters, including 5-HT, NE, and DA (20). Abnormalities of the MAOA gene have been found to be closely related to various neurological and mental disorders, such as behavioural addiction (21–23). Empirical studies have shown that an MAOA gene promoter allele with low activity is associated with pathological gambling (24). Additionally, MAOA-knockout mice have been reported to exhibit impaired nicotine preference but normal response to new stimuli (25). Therefore, MAOA is an important candidate gene for investigating differences in addiction sensitivity among individuals.

According to various studies, 5-HT and MAOA systems play pathophysiological and pathological roles in addictive diseases. We hypothesised that mRNA levels of MAOA and 5-HT_2A_R might be altered in PBMCs of patients with PIU having high rates of self-reported psychiatric symptoms. Therefore, the present study was designed to investigate the mRNA levels of MAOA and 5-HT_2A_R in PBMCs of patients with PIU and healthy controls (HCs). Furthermore, we investigated the association between MAOA and 5-HT_2A_R mRNA levels and comorbid symptoms of patients with PIU.

## Methods

### Participants

The clinical trial was conducted in China from June 2016 to January 2018. In this study, all subjects were screened according to the Beard's Diagnostic Questionnaire for Internet Addiction (26). A total of 49 college students (24 patients with PIU and 25 HCs) were selected for the clinical trial. All the selected students were native Chinese speakers. Inclusion criteria for the PIU group were as follows: (1) meeting the Beard's criteria for internet addiction; (2) aged between 18 and 30 years; (3) not having undergone any form of therapeutic intervention; and (4) not having any other organic or mental illnesses. Overall, 25 HCs were recruited that matched the PIU group in terms of age, gender, and internet age based on the following inclusion criteria: (1) not meeting the Beard's criteria; (2) aged between 18 and 30 years; and (3) not having other organic or mental illnesses. Participants with a history of substance addiction and pregnant or lactating women were excluded from this study.

### Questionnaire

We collected the baseline data, including those for gender, age, internet age, and weekly online time, of the participants through a self-designed questionnaire. Internet age is the actual age of the subject minus the age at which surfing the internet was started. The weekly internet surfing time is an estimate of the weekly internet surfing time of the participant in the past year. In addition, the following scales were used to examine the subjects' comorbid symptoms such as internet addiction, depression, anxiety, sleep quality, and impulsivity:

Young's Internet Addiction Test (IAT) (27) was used to assess the severity of internet addiction. The scale consists of 20 items, which are rated on a 5-point scale (1 = very rarely; 5 = very frequently), ranging from 20 to 100. A high score indicates more severe internet addiction. IAT has been validated in Chinese adolescents, with the Cronbach's alpha of 0.93(28).

Self-Rating Depression Scale (SDS) (29) was used to measure the depression symptoms of patients with PIU. It consists of 20 items, which are rated on a 4-point scale from 1 (never) to 4 (always). The total score was calculated as the sum of the 20 items multiplied by 1.25 and then converted into an integer in the rounded form. The higher the score, the higher is the degree of depression. The Chinese version of the SDS was verified previously (Cronbach's α = 0.75) (30).

Self-Rating Anxiety Scale (SAS) (31) consists of 20 items, which are rated on a 4-point scale (1 = never, 4 =always). A standard score was obtained by multiplying the raw score (the sum of the 20 items) by 1.25. The Chinese version of the SAS was widely used in Chinese samples, with good psychometric characteristics (32).

Pittsburgh Sleep Quality Index (PSQI) (33) was utilised to evaluate the subjective sleep quality over a one-month period. This index consists of 19 items and is used to assess seven components of sleep, namely subjective sleep quality, sleep latency, sleep duration, sleep efficiency, sleep disorders, sleep medication use, and daytime dysfunction. Each item is rated from 0 (no difficulty) to 3 (severe difficulty). The total score is obtained by adding the scores of seven components, ranging from 0 to 21. The higher the total score, the worse is the sleep. In addition, the reliability of the Chinese version of PSQI was verified, with Cronbach's alpha α = 0.81 (34).

Barratt Impulse Scale (BIS-11) (35) was used to assess the impulsivity of participants. The scale contains 30 items, which are rated on a 4-point scale (1 = never; 4 = always), evaluate three dimensions, namely attentional impulsivity, motor impulsivity, and non-planning impulsivity. The higher the score, the higher is the impulsivity. In a previous study, the reliability of the Chinese version of BIS-11 was verified (Cronbach's alpha α = 0.83) (36).

### Mononuclear Cell Separation, Total RNA Isolation, and Reverse Transcription

A total of 5 mL of whole peripheral blood samples were collected into tubes containing ethylene diamine tetra-acetic acid as an anticoagulant. Mononuclear cells were isolated using a gradient centrifuge (Thermo, Waltham, MA, USA). Total RNA was extracted from PBMCs by using Trizol reagent (Invitrogen, Carlsbad, CA, USA). Total RNA was dissolved in 20 μl RNase-free water provided in the kit. The RNA integrity was detected using the Agilent 2200 Bioanalyzer (Agilent, California, USA). Then, 1 μg of total RNA was reverse-transcribed into 20 μl first-strand cDNA by using the Fermentas cDNA synthesis kit (RevertAid™, Fermentas, USA) according to the manufacturer's instruction.

### Real-Time PCR

Nucleotide primers for real-time PCR amplification of beta-actin, MAOA, and 5-HT_2A_R were designed using primer blast software on the National Center for Biotechnology Information website. Primers used for real-time PCR are as follows:

β-actin: forward, 5′-GAAGATCAAGATCATTGCTCCT-3′ and reverse, 5′-TTGCTGATCCACA-3′ (amplicon size, 111-bp).

5-HT_2A_R: forward, 5′-GTAGGTATATCCATGCCAAT-3′ and reverse, 5′-AGGTGATCACCATGATGGTT-3′ (amplicon size, 177-bp).

MAOA: forward, 5′-CTGCCATCATGGGCTT-3′ and reverse, 5′-TTGCTGATCCACA-3′ (amplicon size, 154-bp).

For real-time PCR, the reaction volume was 25 μl/tube, and the following reagents were used: 2x TaqMan Real-time PCR Mix (12.5 μl); upstream primer (10 μm; 0.6 μl); downstream primer (10 μm; 0.6 μl); ddH_2_O (7.7 μl); and cDNA template (3 μl). The reaction was performed on an FTC-3000QPCR system (Funglyn Biotech, Toronto, Canada). Reaction conditions were as follows: pre-denaturing at 95°C for 10 min, denaturation at 95°C for 10 s, annealing at 53°C for 30 s, and 45 cycles of extension for 30 s at 60°C. The beta-actin gene was used as the housekeeping gene for normalising the target gene expression. Relative mRNA levels were calculated using the 2^−Δ*ΔCt*^ method (37).

### Statistical Analysis

All data were analysed using IBM SPSS Statistics 23.0 software (Chicago, USA) and GraphPad Prism 8 Software (California, USA). Shapiro–Wilk test was used to define the normal distribution of variables. Independent-sample *t*-test was performed for continuous variables with normal distribution, and Mann–Whitney *U*-test was performed for continuous variables with non-normal distribution (non-parametric data). Chi-square test was used for categorical variables. The relationships between 5-HT_2A_R and MAOA mRNAs and clinical scores were calculated using Spearman's correlation coefficient. Demographic and clinical characteristics that met normal distribution are presented as mean ± standard deviation (SD), whereas, those lacking normal distribution are expressed as median (inter-quartile range; IQR) or number (%) per group. A *P*-value of <0.05 was considered statically significant.

## Results

### Demographic Information and Clinical Measures

Table 1 shows the comparison of demographics and clinical scores between patients with PIU and HCs. No significant difference in age, gender, or internet age was observed between the two groups (*P* > 0.05). However, the weekly online time was higher in PIU group than in HC group (*P* < 0.001). In addition, consistent with the inclusion results, patients with PIU had higher IAT, SDS, SAS, PSQI, and BIS-11 scores (*P* < 0.001).

**Table 1:** Demographics and clinical characteristics of pathological internet use and healthy control groups.

**Variables**	**Pathological internet use group (*n* = 24)**	**Healthy control group (*n* = 25)**	***P*-value**
Age (years)	21.42 ± 1.20	22.00 (20.00-22.5)	0.82^#^
Gender male (*n*, %)	16 (66.7)	18 (72.0)	0.69^#^
Internet age (years)	8.54 ± 3.12	8.00 (7.00-10.00)	0.93^#^
Internet time (hours/week)	45.00 (36.25-49.75)	19.56 ± 9.84	<0.001^*^
IAT	75.00 ± 6.85	29.52 ± 7.20	<0.001^*^
SDS	57.00 ± 12.86	37.64 ± 8.50	<0.001^*^
SAS	47.00 (41.50-64.50)	34.48 ± 6.65	<0.001^*^
PSQI	9.17 ± 3.16	4.72 ± 3.13	<0.001^*^
BIS-11	79.08 ± 9.52	62.52 ± 9.39	<0.001^*^

*IAT, Young's Internet Addiction Test; SDS, Self Rating Depression Scale; SAS, Self Rating Anxiety Scale; PSQI, Pittsburgh Sleep Quality Index; BIS-11, Barratt Impulse Scale*.

# *Comparison between PIU subjects and healthy controls at baseline, P > 0.05*.

* *Comparison of clinical scores between PIU group and HC group, P < 0.001*.

### Comparisons of MAOA and 5-HT_2A_R mRNA Between Patients With PIU and HCs

**Figure 1:**
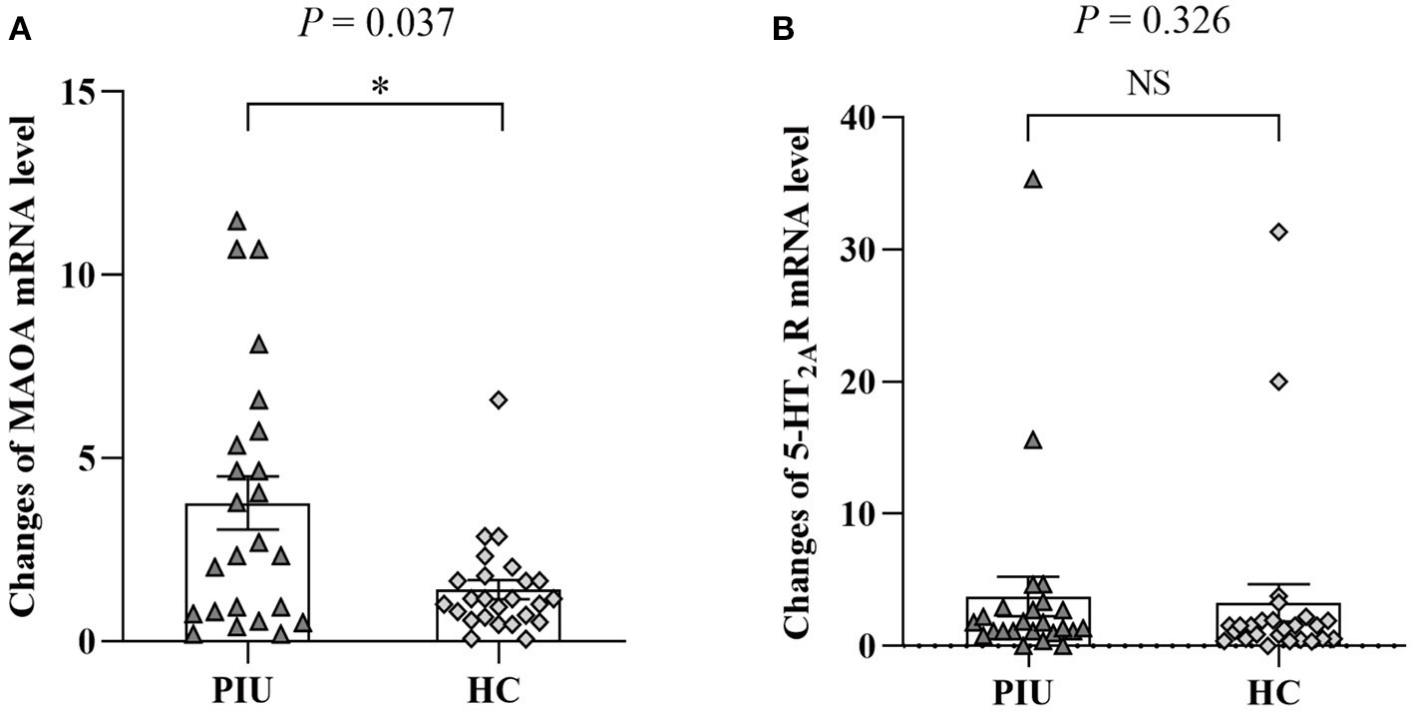
mRNA levels of MAOA **(A)** and 5-HT_2A_R **(B)** in PBMCs of PIU and HC groups. Bars represent means, error bars represent SEM, triangles, and diamonds represent individual data points (*n* = 24 in PIU and 25 in HC groups). *P < 0.05. NS, indicates not statistically.

Figure 1A shows that the mRNA level of MAOA was significantly higher in PBMCs of patients with PIU than in those of HCs (*P* < 0.05). No difference in beta-actin mRNA levels was observed between the two groups.

Figure 1B shows the comparison of the mRNA level of 5-HT_2A_R in PBMCs between patients with PIU and HCs. There was no significant difference in 5-HT_2A_R expression between patients with PIU and HCs (*P* > 0.05).

### Relationship Between MAOA mRNA Levels and Clinical Scores

**Figure 2:**
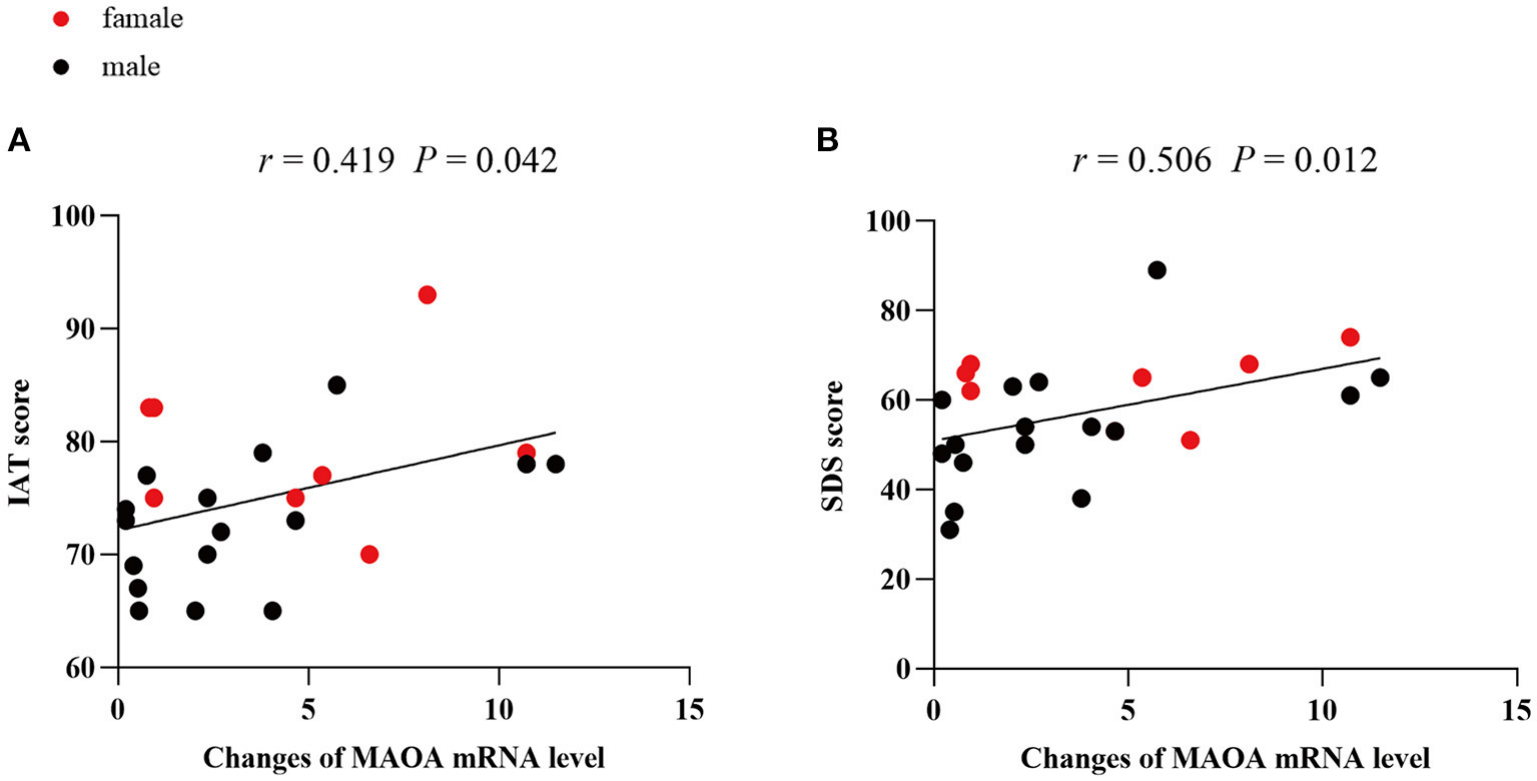
Correlations between mRNA levels of MAOA and clinical scores in PIU. **(A)** Correlation between the mRNA level of MAOA and IAT score of patients with PIU, **(B)** Correlation between the mRNA level of MAOA and SDS score of patients with PIU.

Correlation analyses were conducted (Spearman's *r*) between the relative expression of MAOA mRNA in PBMCs and clinical symptoms data in PIU group. The relative expression of MAOA mRNA was found to be positively correlated to the IAT (*r* = 0.419, *P* = 0.042) and SDS scores (*r* = 0.506, *P* = 0.012) in the PIU group (Figures 2A,B). However, no prominent association was observed between the change in the mRNA level of MAOA and SAS, PSQI, or BIS-11 scores, although, the SAS, PSQI, and BIS-11 scores in patients with PIU were significantly higher than those in HCs.

## Discussion

PIU is a relatively new and complex psychological phenomenon that is related to multiple comorbid symptoms. It has currently become a major public health problem in many countries (38). Various studies have indicated that pathological online behaviour can cause circadian rhythm disorder in patients with PIU, which may include prolonged sleep latency, shortened sleep time, and reduced sleep tendency, leading to sleep disorders, increased fatigue, and daytime sleepiness (39–41). Previous studies have reported that symptoms of depression and anxiety appear to have the most significant correlation with PIU (42). In a systematic review, Carli et al. (43) reported that one of the important underlying causes of depression associated with PIU may be related to sleep disorders caused by excessive internet use. Meanwhile, researchers have also found that patients with PIU are more impulsive and aggressive than those without PIU (44). The results of this study are consistent with those of the aforementioned studies. In addition, PIU has been found to be associated with various substance use disorders, including harmful alcohol use (45, 46). Nevertheless, the neurobiological evidence to confirm these statements is limited.

Serotonin 5-HT_2A_R is widely distributed in the central nervous system. Previous studies have reported that the function and expression level of 5-HT_2A_R were altered in addiction (47–49). However, determining changes in the expression of 5-HT_2A_R in the brain of living patients with behavioural and drug addiction is currently challenging. Based on the peripheral marker hypothesis, some researchers have suggested that changes in the neurotransmitter receptor expression level in the brain may be similar to those in the peripheral blood lymphocytes (50). Therefore, the exploration of the gene expression in peripheral blood is promising. For example, mRNA levels of 5-HT_2A_R in PBMCs from patients with major depression have been reported to be significantly upregulated compared with those in healthy subjects, which is consistent with the reported increase in 5-HT_2A_R binding sites in postmortem brain tissue (51–53). In Alzheimer's disease, a significant decrease in the mRNA level of 5-HT_2A_R in PBMCs was reported (54). We speculated that changes in mRNA level of 5-HT_2A_R in the brain may be reflected in PBMCs of patients with PIU. However, our results indicated no significant difference in the 5-HT_2A_R mRNA levels between PBMCs of patients with PIU and those of HCs. This could indicate that changes in 5-HT_2A_R expression are not involved in PIU. In this regard, studies on the expression level of 5-HT_2A_R during nicotine withdrawal in mice have reported no changes in the density and transcriptional level of 5-HT_2A_R in the midbrain (55). However, morphine tolerance and dependence studies in rats have demonstrated that 5-HT_2A_R is upregulated in the midbrain, pons, medulla oblongata, and amygdala (56). Consequently, the similarity of gene expression patterns between the brain and peripheral blood cells of behaviour addicts should be confirmed in future studies.

MAOA gene is located on the short arm of the X chromosome (Xp11.4–p11.23), and the variable number tandem repeat (VNTR) of the 30-bp repeat unit is present in the promoter region; the number of repetitions is 2–5. Simultaneously, different numbers of tandem repeats form different nucleotide sequences, which affect the transcriptional activity of MAOA (57). MAOA plays an important role in the catabolism of 5-HT, NE, and DA. Changes in these monoamines in the brain are essential for the regulation of motor, cognitive, and emotional functions.

Several psychiatric disorders such as anxiety, depression, alcohol dependence, drug abuse, and aggressive and impulsive behaviours have been found to be related to abnormalities in monoamine oxidase levels (58, 59). Brunner et al. (60) studied MAOA deficiency caused by spontaneous mutation in the MAOA gene in a Dutch family and found that male subjects with MAOA mutation and no activity have a significantly increased level of 5-HT in urine, mild mental retardation, impaired impulse control, and violent aggressive behaviours in response to sudden and stressful stimuli. A study in mice demonstrated that the MAOA gene knockout could elevate the concentrations of 5-HT and NE in the frontal cortex, hippocampus, and cerebellum, causing a considerable increase in fear and aggressive behaviour (61). Notably, the role of 5-HT_2B_ receptor (5-HT_2B_R) gene and MAOA gene in regulating impulsive behaviour may be comparable. Doly et al. (62). found that 5-HT_2B_R gene may be involved in impulsivity by regulating the 5-HT and DA levels. It has been confirmed that the 5-HT_2B_R is located in the dopaminergic neurons that dominate the nucleus accumbens, one of the main components of the brain reward circuit, which is involved in the reinforcement and pleasure generated by substance abuse and addictive behaviour (63, 64). In previous studies on addiction, cannabis-related aggressive behaviour, and cocaine-crack use have been reported to be associated with the 5-HT_2B_R gene (65, 66). HTR2B Q20^*^ carriers showed impulsive and aggressive behaviours, especially under the influence of alcohol (67). Therefore, the 5-HT_2B_R gene may be an important candidate gene for future research on behavioural addiction and substance abuse.

In addition, a study demonstrated that selective monoamine oxidase inhibitors decrease morphine-reinforced behaviour (68). Another study reported that oxycodone-injected mice exhibit higher levels of MAOA mRNA than mice injected with saline (69). Increases in MAOA mRNA level may suggest a high MAOA activity. Elevated MAOA activity in the brain is found in animals exposed to chronic stress and in patients with depression (70, 71). PET research further revealed higher density of MAOA in the brain of individuals with major depression than that in HCs (72). The recurrence of depressive symptoms is related to an increased MAOA density in the brain (73).

To the best of our knowledge, this is the first report on the mRNA level of MAOA in PBMCs of patients with PIU. Consistent with previous studies on major depression, our results demonstrate a remarkably increased MAOA mRNA level in the PIU group. Moreover, we observed that the IAT and SDS scores are positively correlated with the upregulated MAOA mRNA level. Thus, we speculate that the increased MAOA mRNA level might partly represent the underlying neurobiological mechanisms of PIU.

Our study has several limitations. First, it was a single-centre study, and cases were not collected from multiple cities. Second, although, PIU can be divided into sub-types, such as internet gaming disorder, online novel addiction, online gambling addiction, and online shopping addiction, we did not classify each sub-type of PIU; presence of multiple sub-types of internet addiction in individuals may have an impact on the experimental results. Third, the participants were restricted to college students, which limits the promotion of this research. Studies with a larger sample size and inclusion of multiple centres and different age groups along with a careful analysis of the relationship between the PIU and main comorbid symptoms and susceptibility genes will help in overcoming these limitations. In addition, in order to fully understand the relationship between the neurobiological changes of PIU and comorbid symptoms, further, exploration is needed to extend the PIU study to other genes.

## Conclusion

Our study suggests that the increased levels of MAOA mRNA in PBMCs of patients with PIU may be associated with comorbid symptoms, and the increased MAOA mRNA level might be related to the changes in monoamine neurotransmitters. Although, our understanding of mRNA expression of PIU-related genes is inadequate, the present findings provide promising evidence to support that the increased MAOA mRNA level may be a cause of behavioural addiction.

## Data Availability Statement

The original contributions presented in the study are included in the article/supplementary materials, further inquiries can be directed to the corresponding author/s.

## Ethics Statement

The studies involving human participants were reviewed and approved by Sichuan Traditional Chinese Medicine Regional Ethics Review Committee. The patients/participants provided their written informed consent to participate in this study.

## Author Contributions

TZ, HL, and YD conceptualized the study, designed the plan, and managed the project. TZ and HL supervised the study. MQ, CZ, LZ, CW, and YD conducted experiments. MQ, YW, WP, YC, and CW are statistically analyzed. MQ wrote the first draft of the manuscript. All authors read and approved the manuscript.

## Conflict of Interest

The authors declare that the research was conducted in the absence of any commercial or financial relationships that could be construed as a potential conflict of interest.
